# Correction: Zanella et al. Tenofovir, Another Inexpensive, Well Known and Widely Available Old Drug Repurposed for SARS-COV-2 Infection. *Pharmaceuticals* 2021, *14*, 454

**DOI:** 10.3390/ph14080827

**Published:** 2021-08-23

**Authors:** Isabella Zanella, Daniela Zizioli, Francesco Castelli, Eugenia Quiros-Roldan

**Affiliations:** 1 Department of Molecular and Translational Medicine, University of Brescia, 25123 Brescia, Italy; daniela.zizioli@unibs.it; 2 Clinical Chemistry Laboratory, Cytogenetics and Molecular Genetics Section, Diagnostic Department, ASST Spedali Civili di Brescia, Piazzale Spedali Civili 1, 25123 Brescia, Italy; 3 University Department of Infectious and Tropical Diseases, University of Brescia and ASST Spedali Civili di Brescia, Piazzale Spedali Civili 1, 25123 Brescia, Italy; francesco.castelli@unibs.it (F.C.); eugeniaquiros@yahoo.it (E.Q.-R.)

Text Correction

There was an error in the original article [1]. In the Abstract, page 1, line 12 from the top, the sentence “… remdesivir, a nucleotide analog that acts as reverse-transcriptase inhibitor, …” is incorrect. 

A correction has been made to the Abstract.

The incorrect sentence must be replaced with “… remdesivir, a nucleotide analog that acts as RNA-dependent RNA-polymerase inhibitor, …”.

The authors apologize for any inconvenience caused and state that the scientific conclusions are unaffected. The original article has been updated.
